# Tablets of Anatomy

**Published:** 1899-03

**Authors:** 


**Tablets of Anatomy.** By THOMAS COOKE, M.D., and F. G. HAMILTON COOKE. Three parts. [Eleventh Edition.] London: Longmans, Green, and Co. 1898.

These "tablets" are so well known as regards their arrangement and utility that we need not reiterate the many words of praise that have already been bestowed upon them. In Parts I. and II. the newer matter has been interpolated rather than incorporated, and this has led to a somewhat clumsy arrangement of the numbering of the pages and to some unnecessary repetition, which we think might profitably be altered when another edition is provided.

Since the above was written we regret to learn of the death of Mr. Thomas Cooke. While at work in his dissecting room he was seized with an attack of angina pectoris which ended fatally before he could be removed.

